# Thyroid Hormones Correlate with Basal Metabolic Rate but Not Field Metabolic Rate in a Wild Bird Species

**DOI:** 10.1371/journal.pone.0056229

**Published:** 2013-02-20

**Authors:** Jorg Welcker, Olivier Chastel, Geir W. Gabrielsen, Jerome Guillaumin, Alexander S. Kitaysky, John R. Speakman, Yann Tremblay, Claus Bech

**Affiliations:** 1 Norwegian Polar Institute, Tromsø, Norway; 2 Institute of Arctic Biology, University of Alaska Fairbanks, Fairbanks, Alaska, United States of America; 3 Centre d’Etudes Biologiques de Chizé, Centre national de la recherche scientifique, Chizé, France; 4 Institute of Biological and Environmental Sciences, University of Aberdeen, Aberdeen, Scotland, United Kingdom; 5 Institute of Genetics and Developmental Biology, Chinese Academy of Sciences, Beijing, Peoples Republic of China; 6 Centre de Recherche Halieutique Méditerranéenne et Tropicale, Sète cedex, France; 7 Department of Biology, Norwegian University of Science and Technology, Trondheim, Norway; The University of Wollongong, Australia

## Abstract

Thyroid hormones (TH) are known to stimulate *in vitro* oxygen consumption of tissues in mammals and birds. Hence, in many laboratory studies a positive relationship between TH concentrations and basal metabolic rate (BMR) has been demonstrated whereas evidence from species in the wild is scarce. Even though basal and field metabolic rates (FMR) are often thought to be intrinsically linked it is still unknown whether a relationship between TH and FMR exists. Here we determine the relationship between the primary thyroid hormone triiodothyronine (T3) with both BMR and FMR in a wild bird species, the black-legged kittiwake (*Rissa tridactyla*). As predicted we found a strong and positive relationship between plasma concentrations of T3 and both BMR and mass-independent BMR with coefficients of determination ranging from 0.36 to 0.60. In contrast there was no association of T3 levels with either whole-body or mass-independent FMR (R^2^ = 0.06 and 0.02, respectively). In accordance with *in vitro* studies our data suggests that TH play an important role in modulating BMR and may serve as a proxy for basal metabolism in wild birds. However, the lack of a relationship between TH and FMR indicates that levels of physical activity in kittiwakes are largely independent of TH concentrations and support recent studies that cast doubt on a direct linkage between BMR and FMR.

## Introduction

It has long been known that plasma concentrations of thyroid hormones (TH) affect the basal metabolic rate (BMR) of mammals (including humans) and birds [1], [2], [3]. Although evidence from wild animals is still scarce, studies of laboratory animal models have shown stimulating effects of thyroid hormones on tissue oxygen consumption with corresponding effects on BMR [4], [5], [6]. Much less is known about the relationship between thyroid hormones and the field metabolic rate (FMR). However, a strong link between BMR and FMR has often been proposed, particularly during periods of peak energy expenditure such as reproduction [7], [8], [9], leading to the expectation of a positive association between TH and FMR. Yet, the impact of thyroid hormones on FMR of animals remains virtually unknown. In the present study we determined the relationship between the thyroid hormone triiodothyronine (T3) and the basal and field metabolic rates of a free-ranging bird species, the black-legged kittiwake (*Rissa tridactyla*, hereafter called kittiwake).

BMR, which can be seen as the minimal energetic cost of living, is defined as the metabolic rate of a resting, normothermic and post-absorptive adult animal within its thermoneutral zone [10] and has long been proposed to be closely related to concentrations of thyroid hormones in endotherms [4], [11]. In fact, before the advent of radioimmunoassays made it possible to measure plasma concentrations of thyroid hormones directly, BMR was used as a proxy for thyroid status in humans [1], [2]. Today blood concentrations of the two main thyroid hormones, thyroxine (T4) and triiodothyronine (T3) are readily assessed. Although in birds T4 may have high potency for several hormone-triggered responses [12] it is usually regarded as a precursor of the physiologically more active T3. The vast majority of circulating thyroid hormones is bound to plasma proteins and it is the free fraction that is often assumed as physiologically relevant [13].

Although the detailed pathways of how thyroid hormones affect energy metabolism are still debated it is clear that it involves the stimulation of many individual processes in different body tissues [see 4 for a review]. In general, most body tissues have been shown to be thyroid hormone sensitive, and *in vitro* oxygen consumption of a range of tissues is known to differ according to their thyroid status [3], [14], [15]. Hence, many studies on humans and laboratory animals have demonstrated a strong link between thyroid hormones and BMR [4], [6]. For example, early studies demonstrated that thyroidectomy can reduce BMR by up to 40% [4 and references therein], while a positive relationships between TH concentrations and BMR have been reported in euthyroid humans [16], [17]. In the chicken, the correlation coefficient between T3 and BMR was as high as 0.98 [18]. However, for animals under natural conditions much less information is available with respect to the relationship between thyroid hormones and BMR, and results are ambiguous [19], [20], [21], [22].

Similarly, there is a paucity of data with which to evaluate the existence of a link between thyroid hormones and FMR. Even though thyroid hormones have occasionally been used as a proxy for FMR [23], to our knowledge a positive association of thyroid hormones with FMR (or daily energy expenditure) has so far only been demonstrated in humans under controlled conditions [24]. Thermoregulation may be an important driver of FMR, especially in small mammals [25], Given that thyroid hormones stimulate several processes of metabolic heat production and therefore are thought to be important modulators of both obligatory and facultative thermogenesis [5], [12], one may expect a positive relationship of FMR with TH concentrations. However, for species such as kittiwakes that often operate within their thermoneutral zone [26] this is likely to play only a minor role, and physical activity rather than thermoregulation presumably accounts for most of the difference between BMR and FMR. Whether TH concentrations have an effect on levels of physical activity largely remains to be demonstrated [27].

Nonetheless, a relationship between thyroid hormones and FMR is expected under the assumption that BMR and FMR are intrinsically linked, i.e. that the BMR determines the level of FMR an individual can sustain [7], [28], [29]. This hypothesis is mainly based on the notion that a high FMR needs to be sustained by high energy intake and therefore requires large internal organs such as the liver, kidneys and alimentary tract. The size of these organs together with their high metabolic intensities [30] represent an important determinant of BMR and would consequently intrinsically link basal and field metabolic rates [29]. Supporting this hypothesis, a large number of studies have reported a positive association between BMR and FMR both on the inter-specific [9], [29], [31] and intra-specific level [32], [33], [34]. In the present study we tested the strength of the relationship between thyroid hormones (T3) and both BMR and FMR in a free-ranging bird species, the kittiwake during a period of peak energy demands (chick-rearing). We predicted a strong positive relationship between T3 and BMR based on the known contribution of TH to tissue *in vitro* oxygen consumption. We also expected a positive, albeit weaker, association of T3 with FMR given the proposed link between BMR and FMR.

## Materials and Methods

### Ethics Statement

The study was approved by the Governor of Svalbard and by the Norwegian Animal Research Authority.

### Study Area and Animals

Our study was conducted from 21 July to 1 August 2001 and from 23 July to 7 August 2010 in a colony of kittiwakes at Kongsfjorden on the west coast of Spitsbergen, Norway (78°54′N, 12°13′E). The breeding season (May-September) at the study site is characterized by continuous daylight and an average ambient temperature of approx. 4.5°C. Kittiwakes are medium sized (body mass approx 330–400 g), cliff-breeding seabirds that mainly feed on pelagic fish [e.g. 35]. In Spitsbergen, kittiwakes usually lay two eggs and both partners of a pair share parental duties during incubation and chick-rearing. Kittiwakes are sexually size-dimorphic with males weighing on average about 12% more than females [e.g. 36].

### T3 and BMR

In 2001, we captured 24 adult kittiwakes (12 males and 12 females) to simultaneously measure plasma total T3 concentration and BMR. Birds were caught during the chick-rearing period when their chicks were between 2 and 21 d old. Only one adult per nest was captured, and data obtained for males and females were considered as statistically independent. Birds were captured at their nests with a noose at the end of a telescopic pole. Immediately after capture, a blood sample (<500 µl) was collected from the alar vein with a 1 ml heparinized syringe and a 25-gauge needle. Blood samples were stored on ice, centrifuged after max. 10 h and the plasma separated. Plasma samples were then stored at –20°C for triiodothyronine assay (hereafter called ‘T3 in the field’). All birds were weighed to the nearest 2 gram using a Pesola spring balance and the length of the head and bill (‘headbill’) was measured to the nearest 0.5 millimeter. Morphometric measurements were used to determine the sex of the birds following Moe et al. (2002) [36].

After capture and blood sampling, birds were kept in an individual opaque cloth bag to be rapidly (within 15 min) transported by boat to the laboratory to measure BMR by open flow-through respirometry. Birds were fasting for about six hours before being placed into a metabolic chamber. The metabolic chamber was situated inside a larger walk-in temperature-controlled cabinet. The ambient temperature (T_a_, measured using a type T thermocouple placed inside the metabolic chamber) during the measurements was on average 20.7°C (S.D. = 1.6°C, range 18.9–25.5°C), which is within thermoneutrality for kittiwakes [26]. During the experiment, birds were kept in continuous light reflecting natural conditions at the breeding site. Outside air was dried over silica gel and pumped through an approximately 25 L respiratory chamber with a flow rate of approximately 1.0 L/min. Actual flow rates were measured using a calibrated mass flow-meter (Bronkhorst high-tech, type 201C-FA, Ruurlo, the Netherlands). An aliquot of the effluent air was dried over silica-gel and the oxygen concentration measured using an oxygen analyzer (Servomex Xentra 4100, Zoetermeer, the Netherlands). The oxygen analyzer was calibrated using dry outside air (set to 20.95% oxygen) and pure stock nitrogen. The minimum value of oxygen consumption was obtained after an average time of 17 h (S.D. = 4.7, range 10–24 h) after the birds had been captured in the field. The BMR was calculated from the lowest 25-minute running average [10] of instantaneous oxygen consumption. Rates of oxygen consumption (VO_2_) were calculated using formula 3A given by Withers [37], and corrected for wash-out delay in the system by the method described by Niimi (1978) [38]. In this way, we obtained the instantaneous oxygen consumption rates. We assumed a respiratory quotient (RQ) of 0.73. Birds usually have uric acid as their main nitrogenous waste product and because of the stoichiometry of uric acid metabolism RQ during both fasting (predominantly fat metabolism) and during protein metabolism (carbohydrates are negligible in the diet of piscivorous seabirds) will be essentially equal (0.71–0.73). The maximal error in calculating VO_2_ caused by variation in RQ will consequently be small (below 0.5% if RQ varies by ±0.02). BMR (W) was calculated from the value of oxygen consumption rate using a conversion factor of 19.9 J per ml O_2_.

Immediately after the measurements in the respiratory chamber were completed, birds were bled for an additional T3 measurement (hereafter called ‘T3 in the lab’), weighed and released outside the laboratory. All birds were later seen back on their nests without any apparent effect of the handling.

### T3 and FMR

In 2010, we captured 49 adult kittiwakes (24 males and 25 females) to simultaneously measure plasma T3 concentration (total and free fraction) and FMR. This included both partners of 23 breeding pairs and three single birds (1 male and 2 females). Birds were sampled when their chicks were between 18 and 25 d old (hatching dates were known through nest content monitoring every two days). We estimated FMR using the doubly-labeled water (DLW) method [39], [40]. Immediately after capture, birds were weighed and intra-peritoneally injected with 1.25 mL DLW containing 64.0 atom percent excess (APE) oxygen-18 (^18^O) and 36.2 APE deuterium (^2^H). Birds were marked with an individually numbered steel band and a coded plastic band for easy identification. Birds were then kept in a cloth bag for 1 h to allow for complete equilibration of isotopes with the body water of the injected animal [40]. Prior to release, birds were weighed again and an initial blood sample was collected from the alar vein. Blood was stored in several 75 µL glass micro-capillaries which were immediately flame-sealed. Additionally, blood samples were taken from 12 unlabeled adult kittiwakes to determine the mean background level of isotopes [41: method C].

To eliminate high day to day variation of DEE [42], [43], we aimed to recapture all individuals after approximately three days (mean ± SD: 63.7 h±9.9). Upon recapture, birds were weighed and a final blood sample was taken as described above to estimate FMR and measure plasma concentrations of thyroid hormones. We were able to recapture all injected individuals. For thyroid hormone analysis, blood (c. 0.8 mL) was centrifuged, plasma separated and frozen immediately after the sample was taken. The sex of birds in 2010 was determined by molecular sexing following standard procedures as described in Fridolfsson and Ellegren (1999) [44].

Analysis of isotopic enrichment of blood samples was done by isotope ratio mass spectrometry as detailed in Speakman and Krol (2005) [45]. In short, water for analysis of ^2^H and ^18^O was obtained by vacuum distilling blood samples into glass Pasteur pipettes [46]. ^2^H enrichment was determined from hydrogen gas derived from the distilled water by online chromium reduction. ^18^O enrichment was analyzed by equilibration of distilled water with CO_2_-gas of known oxygen isotopic enrichment using the small-sample equilibration technique [47]. Isotope ratios were then determined by gas source isotope mass spectrometry (IRMS) with isotopically characterized gases of H_2_ and CO_2_ in the reference channels. Hydrogen samples were run on an Isoprime 100 mass spectrometer (Isoprime Ltd, Cheadle hulme, UK) and oxygen samples were run on a Micromass µG mass spectrometer (Micromass ltd, Manchester, UK). In both cases the samples were ordered according to their expected enrichment and run from high to low enrichment. The samples were preceded by three characterized working standards whose enrichments had been previously established by reference to the international IAEA standards SMOW and SLAP. These three working standards were run at the start and end of each batch of samples. In the initial standard set the order was low to high, this was followed by the samples running high to low, and then the final batch of standards was also run low to high. This ordering minimized large isotope changes between samples to minimize memory effects. The measured enrichments of the standards were compared to their established enrichments to generate a correction at the start and end of each run. This allowed us to correct for machine drift throughout the measurements of each batch of samples. A typical run would include 30–40 samples (15 to 20 duplicates) bracketed between the standards which were run in quintuplicate. Enrichment of the injectate was estimated by a dilution series with tap water and mass spectrometric analysis of 5 subsamples of each solution [45].

We calculated rates of CO_2_-production using a single pool model as recommended for this size of animal [40], [48]. We corrected for fractionation effects assuming a fixed evaporation of 25% of the total water efflux [equation 7.17, 40] which has been shown in several studies to minimize deviations from reference methods [45], [49], [50]. Initial body water was determined from the ^18^O dilution space which was calculated by the plateau method [40]. Final body water was inferred from final body mass and assuming a similar fraction of body water throughout the measurement period.

The rate of CO_2_-production was converted into estimates of FMR (W) using a caloric equivalent of 27.639 J mL CO_2_^−1^. This represents the mean conversion factor derived from year-specific factors based on dietary information and estimated over 5 study years for kittiwakes at the same colony [51]. Variation among year-specific conversion factors was negligible [CV: 0.08%, 51].

### T3 Radioimmunoassays

In 2001, plasma concentrations of total 3, 3′-Triiodo-L-Thyronine (tT3) were determined by radioimmunoassay at the Centre d’Etudes Biologiques de Chizé as detailed in Chastel et al. [20]. All samples were analyzed in a single assay and the intra-assay coefficient of variation (CV) was 3% (n = 4 duplicates).

In 2010, we determined both tT3 and unbound (free) T3 (fT3) concentrations at the Institute of Arctic Biology, Fairbanks, by radioimmunoassay based on a commercially available kit (MP Biomedicals) optimized for our study species. The dose-response curve of pooled kittiwake plasma was parallel with standard curves of both tT3 and fT3 assay kits. All samples were analyzed in duplicate. Total T3 was analyzed in two assays with a CV of 2.0% and 6.5% for intra- and inter-assay variability, respectively. Free T3 was analyzed in a single assay with an intra-assay CV of 2.3%.

### Data Analysis

To check for potential confounding effects of diurnal variation [52], ambient temperature [53] and handling time [54] on T3, we plotted T3 values against time of the day when the sample was collected (range: 10h30–23h30), ambient air temperature recorded at the colony (range: 1.6–7.8°C, 2001 only) and handling time during blood sampling (range: 1–10 min). No significant relationships were found (linear regressions, all P>0.11, polynomial regression for time of day, p>0.5; see also [10]). Similarly, we tested for an effect of deviations of the sampling period from a multiple of 24 h periods on FMR. As has previously been shown for birds breeding in the continuous daylight of the Polar summer [51], [55], we did not find support for a diurnal rhythm of FMR in kittiwakes (*F*_1,47_ = 0.01, *P* = 0.97).

Plasma concentrations of tT3 were significantly higher in 2010 compared to 2001 (*F*_1,71_ = 73.3, *P*<0.001, see table 1). This difference may reflect inter-annual variation or may partly be driven by differences in the radioimmunoassays used (see above). Hence, tT3 data from the different study years were analyzed separately.

**Table 1:** Mean (±SE) body mass, plasma levels of T3 and metabolic rates of chick-rearing of male and female kittiwakes in Kongsfjorden, Svalbard. 12 males and 12 females were sampled in 2001, 24 females and 25 males were sampled in 2010.

	Males	Females	*F_1,22_*	*P*
*2001*				
Body mass colony (g)	384.58±5.72	338.33±7.72	23.2	<0.001
Body mass lab (g)	347.50±4.40	311.42±5.87	24.2	<0.001
T3 colony (ng/ml)	1.64±0.22	1.25±0.09	1.4	0.24
T3 lab (ng/ml)	1.17±0.14	1.31±0.21	0.1	0.74
BMR (Watt)	2.61±0.11	2.41±0.10	1.8	0.19
*2010*				
Body mass (g)	394.21±5.25	343.10±3.31	68.9	<0.001
Total T3 (ng/ml)	3.18±0.23	2.81±0.17	1.5	0.23
Free T3 (pg/ml)	7.20±0.77	5.44±0.52	2.6	0.11
FMR (Watt)	12.48±0.52	7.92±0.35	51.5	<0.001

We used ordinary least squares regressions to determine the relationship of BMR with tT3 measured in the field and the lab. To evaluate the effect of sex on these relationships, sex was included as an additional factor in a second set of models. BMR was significantly positively related with body mass (*F*_1,22_ = 15.74, *P*<0.001, *R^2^* = 0.42). To remove the effect of body mass, we ran a second set of models on mass-independent BMR which was calculated as the residuals of the regression of BMR on body mass.

As both partners of breeding pairs were sampled in 2010, estimates of FMR of partners were not statistically independent. We therefore started to determine the relationship of FMR with total and free T3 by fitting general linear mixed effects models containing ‘nest identity’ as a random effect. However, in all cases the random variance component was estimated to be very close to zero indicating that the variability between individuals of different pairs was not larger than expected from within-pair variability. Hence, in our data partners of a pair can be regarded as statistically independent with respect to FMR and consequently we fitted ordinary least-squares models to those data. Similarly to BMR, in a second step we removed the effect of body mass by calculating mass-independent FMR as above.

Model fits were assessed by examining diagnostic plots; all hormone data and FMR were log-transformed to meet the condition of normality. All statistical analyses were performed using R.15.0 [56].

## Results

In 2001, tT3 levels measured in the lab were positively and significantly related to those measured at the colony (*F*_1, 22_ = 11.12, *P* = 0.003, *R^2^* = 0.34), and mean tT3 concentrations were similar at both sampling events (*t* = 1.43, N = 24, *P* = 0.16). In addition, plasma levels of tT3 measured at the colony and in the lab were similar between males and females (Table 1). Similarly, both BMR and mass-independent BMR did not differ between males and females (Table 1). In addition, there was a significant positive relationship between plasma levels of tT3 and body mass measured at the colony (*F*_1,22_ = 9.87, *P* = 0.005, *R^2^* = 0.31) whereas such a relationship was absent for tT3 levels and body mass measured in the lab (*F*_1,22_ = 0.80, *P* = 0.38, *R^2^* = 0.04).

**Figure 1:**
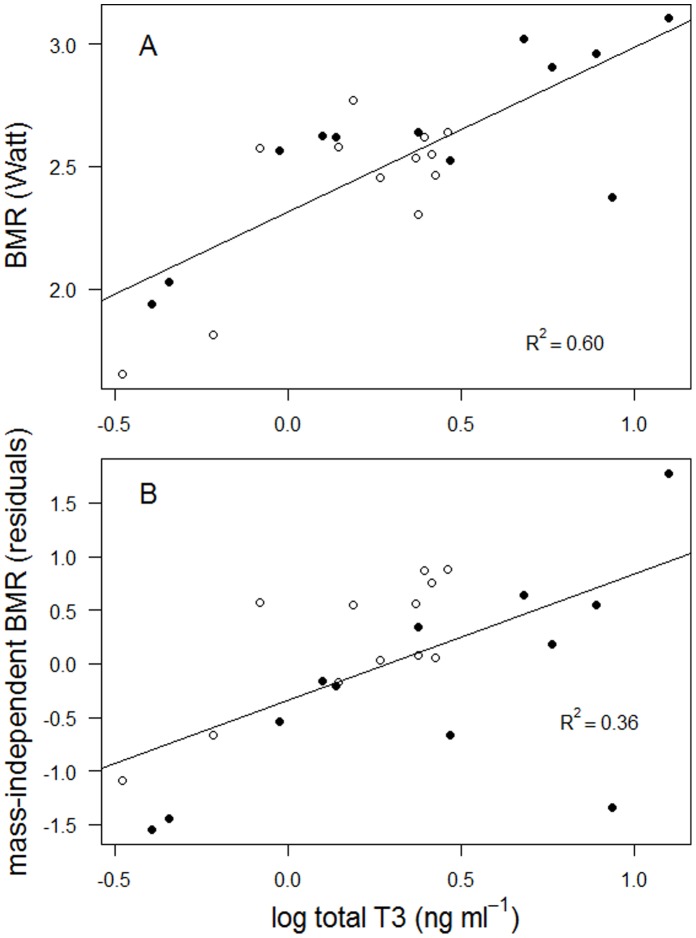
Relationship between plasma levels of total T3 and BMR (panel *A*), and mass-independent BMR (residuals of BMR regressed on body mass, panel *B*) in chick-rearing black-legged kittiwakes in Kongsfjorden, Svalbard in 2001. T3 was measured in blood samples obtained at the colony prior to BMR measurements in the lab using a respiratory chamber. Open symbols denote females and closed symbols denote males.

There were strong positive relationships between tT3 and BMR (Fig. 1). Total T3 measured in the field explained 60.1% of the observed variation in BMR (Fig. 1; *F*_1,22_ = 33.19, *P*<0.001), tT3 measured in the lab explained 36.3% of the variation (*F*_1,22_ = 12.57, *P* = 0.002). These relationships persisted when body mass was accounted for (mass-independent BMR, Fig. 1; *F*_1,22_ = 11.72, *P* = 0.002, *R^2^* = 0.35) and tT3 measured in the lab (*F*_1,22_ = 14.56, *P*<0.001, *R^2^* = 0.40). Accounting additionally for the effect of body mass on T3 in the field also had no strong effect on the relationship (*F*_1,22_ = 10.09, *P* = 0.004, *R^2^* = 0.31). Together, T3 and body mass explained 68.9% and 65.8% of the variation in BMR for T3 measured in the field and in the lab, respectively.

In accordance with results from the first study year, in 2010 male kittiwakes weighed approx. 14% more compared to females (Table 1), while there was no sex difference between plasma concentrations of both total and free T3 (Table 1). In contrast to BMR, FMR of males exceeded those of females by 57.6% (Table 1), and the effect of sex was not completely removed when accounting for body mass (*F*_1,47_ = 4.646, *P* = 0.036). Plasma concentrations of total and free T3 were highly correlated (*F*_1,47_ = 162.7, *P*<0.001, *R^2^* = 0.78). Furthermore, in 2010 there was no effect of body mass on either total (*F*_1,47_ = 2.12, *P* = 0.15, *R^2^* = 0.04) or free T3 (*F*_1,47_ = 3.40, *P*<0.07, *R^2^* = 0.08), while FMR increased significantly with body mass (*F*_1,47_ = 32.29, *P*<0.001, *R^2^* = 0.41).

**Figure 2:**
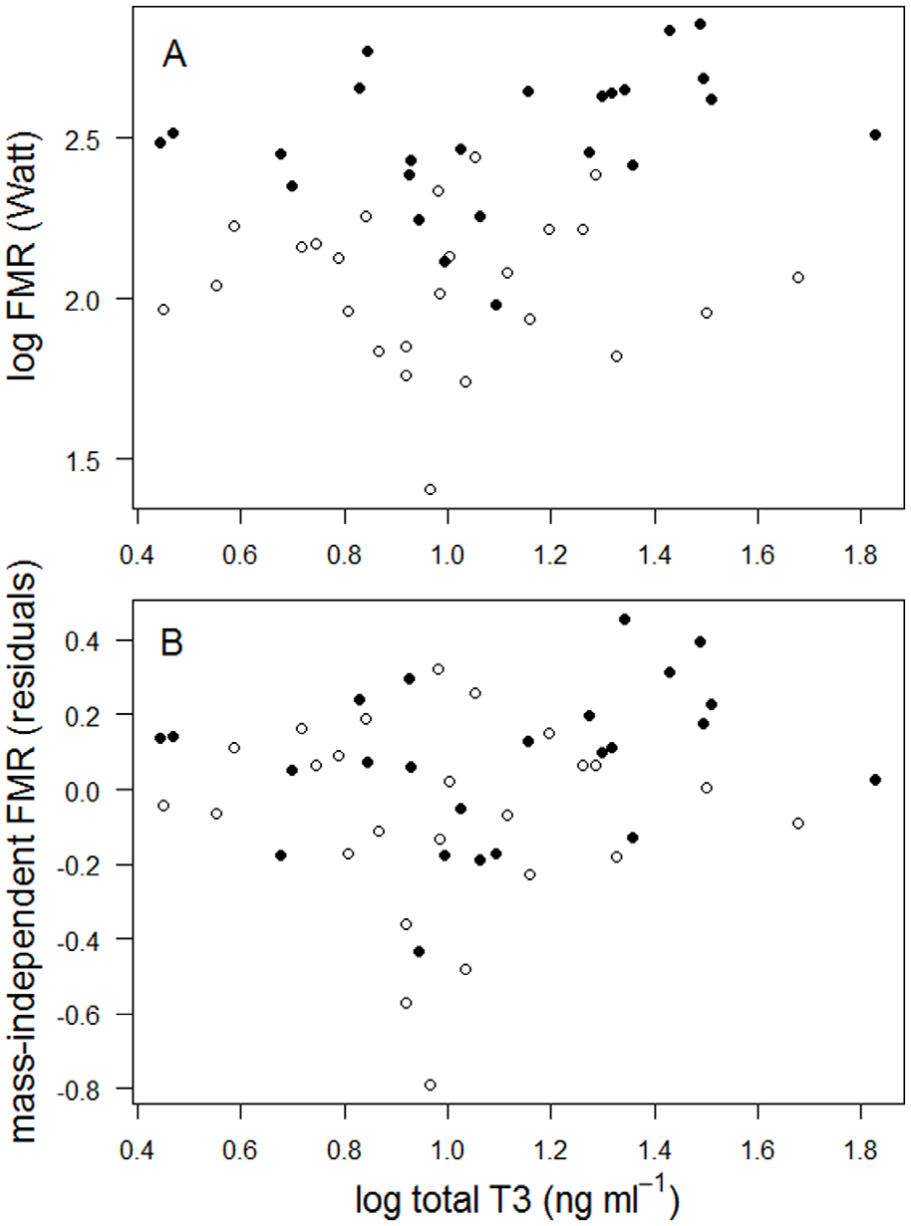
Relationship between plasma levels of total T3 and FMR (panel *A*), and mass-independent FMR (residuals of FMR (log) regressed on body mass, panel *B*) in chick-rearing black-legged kittiwakes in Kongsfjorden, Svalbard in 2010. Open symbols denote females and closed symbols denote males.

In contrast to BMR there was no strong relationship between either FMR or mass-independent FMR with tT3 (Fig. 2; FMR: *F*_1,47_ = 2.98, *P* = 0.09, *R^2^* = 0.06; mass-independent FMR: *F*_1,47_ = 1.01, *P* = 0.32, *R^2^* = 0.02). Similarly, relationships between free T3 and field metabolic rates were weak and statistically non-significant (FMR: *F*_1,47_ = 3.90, *P* = 0.054, *R^2^* = 0.08; mass-independent FMR: *F*_1,47_ = 0.99, *P* = 0.32, *R^2^* = 0.02).

## Discussion

Even though many *in vitro* studies have shown the stimulating effect of TH on tissue metabolic rate and the link between TH and BMR is well established in humans and several model species in the lab, evidence for a similar relationship in free-ranging animals is still scarce. Our data show a strong positive correlation between the thyroid hormone T3 and BMR in wild-caught kittiwakes suggesting that the linkage demonstrated in laboratory animals also holds under natural conditions. In kittiwakes it has previously been shown that adjustments of BMR across life history stages are at least partly attained through modulation of the metabolic intensity of internal organs such as the kidneys accompanied by corresponding changes in TH [57], and hence it seems likely that TH is involved in that process. This result is also in accordance with two previous studies on free-ranging house sparrows (*Passer domesticus*) [20] and red knots (*Calidris canutus*) [21] that both reported similarly strong relationships between TH and BMR but contrasts with findings from Burger and Denver (2002) [19] for northern cardinals (*Cardinalis cardinalis*). However, in the latter study, hormone concentrations and metabolic rates were not measured in the same individuals and a relationship between TH and BMR across sampling locations might have been obscured by several confounding factors such as ambient temperature, food intake, time of day or other environmental stimuli that are known to affect thyroid function [4], [52], [53], [58].

BMR is difficult to measure in the field and might, especially in species sensitive to handling, lead to spurious results [59]. Although it is still debated whether variation in BMR is the cause or the consequence of variation in TH concentrations [1], the strength of the relationship of TH with BMR within species, as demonstrated in our and previous studies [20], and the fact that this relationship was independent of body mass suggests that T3 might serve as a proxy for BMR in free-ranging animals. However, further research on a larger variety of species is warranted to determine whether this conclusion can be generalized or only holds for kittiwakes during the breeding season.

It is generally assumed that TH concentrations are related to the metabolic intensity of a body tissue rather than the overall metabolic rate of an organ. Therefore, we would have expected TH levels to be more strongly related to mass-independent BMR rather than whole-body BMR. However, in our data whole-body and mass-independent BMR were highly correlated and differences in their relationship with TH concentrations were minor. Larger datasets are needed to evaluate potential differences in the relationships of TH with whole-body and mass-independent metabolic rates.

We measured TH concentrations twice in each individual, in the field prior to BMR measurements and in the lab immediately after measurement of BMR. This was mainly done to rule out a possible effect of handling on T3 titres potentially obscuring the link with BMR [54]. However, tT3 in the field and tT3 in the lab were highly correlated and means did not differ significantly. This indicates that the possible impact of handling and BMR procedures on tT3 levels after the BMR measurements was negligible. As it is known that plasma TH concentrations increase within minutes after an immediate stressor such as our handling routines [54; A. Kitaysky unpublished data] it is likely that during the extended time birds spent in the respiratory chamber, tT3 levels returned to previous ‘baseline’ levels. The short half-life of TH in birds [12] may facilitate the apparent resilience of TH concentrations in kittiwakes. As a result, the relationships of BMR with tT3 before and after BMR measurements were similarly strong.

In contrast to BMR, both tT3 and fT3 were only weakly and non-significantly related to the FMR of kittiwakes. To date, surprisingly few attempts have been made to evaluate the relationship between TH and FMR, and these attempts are restricted to humans and laboratory animals. While under controlled conditions, TH concentrations of humans were positively related to daily energy expenditure [24], no such relationship was evident in unrestricted humans [60]. In addition, treatment with exogenous T4 increased the standard metabolic rate of lizards by almost 60% whereas the effect on DEE was negligible [61].

Estimates of energy expenditure derived by the DLW method have been shown to closely correspond to reference methods such as indirect calorimetry. Deviation from reference methods is particularly small for mean estimates of FMR for groups of animals; estimates for single individuals are known to differ to a larger extent [40]. If the stochastic error in FMR estimates exceeds those of BMR measurements, the strength of the relationship of TH with FMR can be expected to be reduced in comparison to BMR. However, we regard it as unlikely that a possible difference in stochastic error can explain the distinct difference between the strong relationship of TH with BMR and the absence of a relationship with FMR, respectively. In addition, plasma tT3 levels in 2010 were increased twofold in comparison to 2001 (see Methods). As TH concentrations are known to be highly variable both within and among individuals [62] this differences may reflect natural inter-annual variability of tT3 in kittiwakes. Yet, although low intra- and inter-assay variation in radioimmunoassays indicate that TH concentrations were assessed reliably, we cannot exclude the possibility that this difference was at least partly caused by differences in methodology between years. However, as we did not attempt to compare TH concentrations between years, our within-year correlations of tT3 with metabolic rates remain unaffected by inter-annual variability.

The lack of a relationship of TH with FMR might be unexpected because FMR and BMR are often thought to be intrinsically linked [9]. This is based on the idea that high levels of physical exercise require a large metabolic machinery to sustain such exercise levels, and the maintenance costs of these large machinery/organs would cause a close association between FMR and BMR. However, while an association of BMR with FMR has repeatedly been demonstrated [9], [32], [33] increasing evidence puts into question the ubiquity and nature of this relationship [8], [63], [64], [65], [66]. For example, Speakman et al. (2003) [66] suggested that an apparent relationship between FMR and BMR in free-ranging voles was driven by covariation in response to extrinsic factors rather than intrinsic coupling. Also, work on laboratory mice revealed no evidence for a strong relationship between organ size and BMR, a critical assumption underlying an intrinsic link between BMR and FMR. In kittiwakes, Bech et al (2002) [67] proposed that FMR and BMR might be adjusted independently within a reproductive season making an intrinsic association of the two metabolic rates unlikely. Even though we did not measure FMR and BMR in the same individuals, the strength of the relationship of TH with BMR and the lack of such a relationship with FMR supports this view.

A positive correlation of TH with FMR could also be expected if TH had a direct effect on levels of physical activity of an animal, as physical activity is likely to account for most of the difference between BMR and FMR in kittiwakes. However, while it has been shown that hyperthyroidism caused by high doses of exogenous TH increased physical activity or caused hyperactivity in laboratory rats [27], [68], an effect of natural variability of TH concentrations on physical activity is yet to be demonstrated. In accordance with this, our results cast doubt on thyroid hormones as an intrinsic modulator of FMR in free-living animals and questions attempts to use plasma concentrations of TH as a proxy for FMR in field studies [23].

## References

[1] Hulbert AJ, Else PL (2004) Basal metabolic rate: History, composition, regulation, and usefulness. Physiological and Biochemical Zoology 77: 869–876. 15674762 10.1086/422768

[2] Du Bois EF (1936) Basal metabolism in health and disease. Philadelphia: Lea & Febinger.

[3] Barker SB (1951) Mechanism of action of the thyroid hormone. Physiological Reviews 31: 205–243. 14853652 10.1152/physrev.1951.31.3.205

[4] Hulbert AJ (2000) Thyroid hormones and their effects: a new perspective. Biological Reviews 75: 519–631. 11117200 10.1017/s146479310000556x

[5] Silvestri E, Schiavo L, Lombardi A, Goglia F (2005) Thyroid hormones as molecular determinants of thermogenesis. Acta Physiologica Scandinavica 184: 265–283. 16026419 10.1111/j.1365-201X.2005.01463.x

[6] Kim B (2008) Thyroid hormone as a determinant of energy expenditure and the basal metabolic rate. Thyroid 18: 141–144. 18279014 10.1089/thy.2007.0266

[7] Hammond KA, Diamond J (1997) Maximal sustained energy budgets in humans and animals. Nature 386: 457–462. 9087402 10.1038/386457a0

[8] Careau V, Reale D, Garant D, Pelletier F, Speakman JR, et al. (2012: in press) Resting metabolic rate and daily energy expenditure are correlated in wild chipmunks, but only during reproduction. Journal of Experimental Biology. 10.1242/jeb.076794 23077163

[9] Daan S, Masman D, Groenewold A (1990) Avian basal metabolic rates - their association with body composition and energy expenditure in nature. American Journal of Physiology 259: R333–R340. 2386245 10.1152/ajpregu.1990.259.2.R333

[10] Bech C, Langseth I, Gabrielsen GW (1999) Repeatability of basal metabolism in breeding female kittiwakes *Rissa tridactyla* . Proceedings of the Royal Society B: Biological Sciences 266: 2161–2167.

[11] Freake HC, Oppenheimer JH (1995) Thermogenesis and thyroid function. Annual Review of Nutrition 15: 263–291. 10.1146/annurev.nu.15.070195.001403 8527221

[12] McNabb FMA (2000) Thyroids. In: Whittow GC, editor. Avian Physiology. San Diego: Academic Press. 461–471.

[13] Mendel CM (1989) The free hormone hypothesis - a physiologically based mathematical model. Endocrine Reviews 10: 232–274. 2673754 10.1210/edrv-10-3-232

[14] Barker SB, Klitgaard HM (1952) Metabolism of tissues excised from thyroxine-injected rats. American Journal of Physiology 170: 81–86. 12985867 10.1152/ajplegacy.1952.170.1.81

[15] Harper ME, Brand MD (1993) The quantitative contributions of mitochondrial proton leak and ATP turnover reactions to the changed respiration rates of hepatocytes from rats of different thyroid status. Journal of Biological Chemistry 268: 14850–14860. 8392060

[16] Stenlof K, Sjostrom L, Fagerberg B, Nystrom E, Lindstedt G (1993) Thyroid hormones, procollagen-III peptide, body-composition and basal metabolic rate in euthyroid individuals. Scandinavian Journal of Clinical & Laboratory Investigation 53: 793–803. 8140389 10.3109/00365519309086491

[17] Johnstone AM, Murison SD, Duncan JS, Rance KA, Speakman JR (2005) Factors influencing variation in basal metabolic rate include fat-free mass, fat mass, age, and circulating thyroxine but not sex, circulating leptin, or triiodothyronine. American Journal of Clinical Nutrition 82: 941–948. 16280423 10.1093/ajcn/82.5.941

[18] Bobek S, Jastrzebski M, Pietras M (1977) Age-related changes in oxygen-consumption and plasma thyroid hormone concentration in young chicken. General and Comparative Endocrinology 31: 169–174. 844676 10.1016/0016-6480(77)90014-4

[19] Burger MF, Denver RJ (2002) Plasma thyroid hormone concentrations in a wintering passerine bird: Their relationship to geographic variation, environmental factors, metabolic rate, and body fat. Physiological and Biochemical Zoology 75: 187–199. 12024294 10.1086/338955

[20] Chastel O, Lacroix A, Kersten M (2003) Pre-breeding energy requirements: thyroid hormone, metabolism and the timing of reproduction in house sparrows Passer domesticus. Journal of Avian Biology 34: 298–306.

[21] Vezina F, Gustowska A, Jalvingh KM, Chastel O, Piersma T (2009) Hormonal Correlates and Thermoregulatory Consequences of Molting on Metabolic Rate in a Northerly Wintering Shorebird. Physiological and Biochemical Zoology 82: 129–142. 19199554 10.1086/596512

[22] Li YG, Yan ZC, Wang DH (2010) Physiological and biochemical basis of basal metabolic rates in Brandt’s voles (Lasiopodomys brandtii) and Mongolian gerbils (Meriones unguiculatus). Comparative Biochemistry and Physiology a-Molecular & Integrative Physiology 157: 204–211. 10.1016/j.cbpa.2010.06.183 20601053

[23] Duriez O, Pastout-Lucchini L, Boos M, Chastel O, Fritz H, et al (2004) Low levels of energy expenditure in a nocturnal, forest-dwelling wader, the Eurasian Woodcock Scolopax rusticola. Ardea 92: 31–42.

[24] Toubro S, Sorensen TIA, Ronn B, Christensen NJ, Astrup A (1996) Twenty-four-hour energy expenditure: The role of body composition, thyroid status, sympathetic activity, and family membership. Journal of Clinical Endocrinology & Metabolism 81: 2670–2674. 8675595 10.1210/jcem.81.7.8675595

[25] Westerterp KR, Speakman JR (2008) Physical activity energy expenditure has not declined since the 1980s and matches energy expenditures of wild mammals. International Journal of Obesity 32: 1256–1263. 18504442 10.1038/ijo.2008.74

[26] Gabrielsen GW, Mehlum F, Karlsen HE (1988) Thermoregulation in 4 species of arctic seabirds. Journal of Comparative Physiology B-Biochemical Systemic and Environmental Physiology 157: 703–708.

[27] Levine JA, Nygren J, Short KR, Nair KS (2003) Effect of hyperthyroidism on spontaneous physical activity and energy expenditure in rats. Journal of Applied Physiology 94: 165–170. 12486020 10.1152/japplphysiol.00499.2002

[28] Weiner J (1992) Physiological limits to sustainable energy budgets in birds and mammals - ecological impliactions. Trends in Ecology & Evolution 7: 384–388. 21236073 10.1016/0169-5347(92)90009-Z

[29] Speakman JR (2000) The cost of living: Field metabolic rates of small mammals. Advances in Ecological Research 30: 177–297.

[30] Rolfe DFS, Brown GC (1997) Cellular energy utilization and molecular origin of standard metabolic rate in mammals. Physiological Reviews 77: 731–758. 9234964 10.1152/physrev.1997.77.3.731

[31] White CR, Seymour RS (2004) Does basal metabolic rate contain a useful signal? Mammalian BMR allometry and correlations with a selection of physiological, ecological, and life-history variables. Physiological and Biochemical Zoology 77: 929–941. 15674767 10.1086/425186

[32] Daan S, Masman D, Strijkstra A, Verhulst S (1989) Intraspecific allometry of basal metabolic rate - relations with body size, temperature, composition, and circadian phase in the kestrel, *Falco tinnunculus* . Journal of Biological Rhythms 4: 267–283. 2519593

[33] Nilsson JA (2002) Metabolic consequences of hard work. Proceedings of the Royal Society of London Series B-Biological Sciences 269: 1735–1739. 10.1098/rspb.2002.2071 PMC1691085 12204136

[34] Tieleman BI, Dijkstra TH, Klasing KC, Visser GH, Williams JB (2008) Effects of experimentally increased costs of activity during reproduction on parental investment and self-maintenance in tropical house wrens. Behavioral Ecology 19: 949–959.

[35] Barrett RT (2007) Food web interactions in the southwestern Barents Sea: black-legged kittiwakes *Rissa tridactyla* respond negatively to an increase in herring Clupea harengus. Marine Ecology Progress Series 349: 269–276.

[36] Moe B, Langseth I, Fyhn M, Gabrielsen GW, Bech C (2002) Changes in body condition in breeding Kittiwakes *Rissa tridactyla* . Journal of Avian Biology 33: 225–234.

[37] Withers PC (1977) Measurement of VO2, VCO2, and evaporative water-loss with a flow-through mask. Journal of Applied Physiology 42: 120–123. 833070 10.1152/jappl.1977.42.1.120

[38] Niimi AJ (1978) Lag adjustment between estimated and actual physiological responses conducted in flow-through systems. Journal of the Fisheries Research Board of Canada 35: 1265–1269.

[39] Butler PJ, Green JA, Boyd IL, Speakman JR (2004) Measuring metabolic rate in the field: the pros and cons of the doubly labelled water and heart rate methods. Functional Ecology 18: 168–183.

[40] Speakman JR (1997) Doubly labelled water - theory and practice. London: Chapman & Hall.

[41] Speakman JR, Racey PA (1987) The equilibrium concentration of O-18 in body-water: implications for the accuracy of the doubly-labeled water technique and a potential new method of measuring RQ in free-living animals. Journal of Theoretical Biology 127: 79–95.

[42] Berteaux D, Thomas DW, Bergeron JM, Lapierre H (1996) Repeatability of daily field metabolic rate in female meadow voles (*Microtus pennsylvanicus*). Functional Ecology 10: 751–759.

[43] Speakman JR, Racey PA, Haim A, Webb PI, Ellison GTH, et al (1994) Interindividual and intraindividual variation in daily energy expenditure of the pouched mouse (*Saccostomus campestris*). Functional Ecology 8: 336–342.

[44] Fridolfsson AK, Ellegren H (1999) A simple and universal method for molecular sexing of non-ratite birds. Journal of Avian Biology 30: 116–121.

[45] Speakman JR, Krol B (2005) Comparison of different approaches for the calculation of energy expenditure using doubly labeled water in a small mammal. Physiological and Biochemical Zoology 78: 650–667. 15957119 10.1086/430234

[46] Nagy KA (1983) The doubly labelled water (^3^HH^18^O) method: a guide to its use. UCLA Publication, University of California, Los Angeles.

[47] Speakman JR, Nagy KA, Masman D, Mook WG, Poppitt SD, et al (1990) Interlaboratory comparison of different analytical techniques for the determination of O-18 abundance. Analytical Chemistry 62: 703–708.

[48] Speakman JR (1993) How should we calculate CO2 production in doubly labelled water studies of animals. Functional Ecology 7: 746–750.

[49] Van Trigt R, Kerstel ERT, Neubert REM, Meijer HAJ, McLean M, et al (2002) Validation of the DLW method in Japanese quail at different water fluxes using laser and IRMS. Journal of Applied Physiology 93: 2147–2154. 12433938 10.1152/japplphysiol.01134.2001

[50] Visser GH, Schekkerman H (1999) Validation of the doubly labeled water method in growing precocial birds: The importance of assumptions concerning evaporative water loss. Physiological and Biochemical Zoology 72: 740–749. 10603338 10.1086/316713

[51] Welcker J, Moe B, Bech C, Fyhn M, Schultner J, et al (2010) Evidence for an intrinsic energetic ceiling in free-ranging kittiwakes *Rissa tridactyla* . Journal of Animal Ecology 79: 205–213. 19817918 10.1111/j.1365-2656.2009.01626.x

[52] Klandorf H, Sharp PJ, Duncan IJH (1978) Variations in levels of plasma thyroxine and triiodothyronine in juvenile female chicken during 24-hr and 16-hr lighting cycles. General and Comparative Endocrinology 36: 238–243. 738598 10.1016/0016-6480(78)90029-1

[53] Dawson WR, Carey C, Vanthof TJ (1992) Metabolic aspects of shivering thermogenesis in passerines during winter. Ornis Scandinavica 23: 381–387.

[54] Gratto-Trevor CL, Oring LW, Fivizzani AJ (1991) Effects of blood-sampling stress on hormone levels in the semipalmated sandpiper. Journal of Field Ornithology 62: 19–27.

[55] Welcker J, Harding AMA, Kitaysky AS, Speakman JR, Gabrielsen GW (2009) Daily energy expenditure increases in response to low nutritional stress in an Arctic-breeding seabird with no effect on mortality. Functional Ecology 23: 1081–1090.

[56] R Development Core Team (2012) R: A language and environment for statistical computing. Vienna, Austria: R Foundation for Statistical Computing.

[57] Rønning B, Moe B, Chastel O, Broggi J, Langset M, et al (2008) Metabolic adjustments in breeding female kittiwakes (*Rissa tridactyla*) include changes in kidney metabolic intensity. Journal of Comparative Physiology B-Biochemical Systemic and Environmental Physiology 178: 779–784. 10.1007/s00360-008-0268-6 18437391

[58] Eales JG (1988) The influence of nutritional state on thyroid function in various vertebrates. American Zoologist 28: 351–362.

[59] Ellis HI, Gabrielsen GW (2002) Energetics of free-ranging seabirds. In: Schreiber EA, Burger J, editors. Biology of marine birds. Boca Raton: CRC Press.

[60] Starling RD, Toth MJ, Carpenter WH, Matthews DE, Poehlman ET (1998) Energy requirements and physical activity in free-living older women and men: a doubly labeled water study. Journal of Applied Physiology 85: 1063–1069. 9729584 10.1152/jappl.1998.85.3.1063

[61] Joos B, John-Alder HB (1990) Effects of thyroxine on standard and total metabolic rates in the lizard *Sceloporus undulatus* . Physiological Zoology 63: 873–885.

[62] Corbel H, Morlon F, Groscolas R (2008) Is fledging in king penguin chicks related to changes in metabolic or endocrinal status? General and Comparative Endocrinology 155: 804–813. 18155218 10.1016/j.ygcen.2007.11.006

[63] Fyhn M, Gabrielsen GW, Nordoy ES, Moe B, Langseth I, et al (2001) Individual variation in field metabolic rate of kittiwakes (*Rissa tridactyla*) during the chick-rearing period. Physiological and Biochemical Zoology 74: 343–355. 11331506 10.1086/320419

[64] Meerlo P, Bolle L, Visser GH, Masman D, Daan S (1997) Basal metabolic rate in relation to body composition and daily energy expenditure in the field vole, Microtus agrestis. Physiological Zoology 70: 362–369. 9231410 10.1086/639616

[65] Speakman JR, Krol E, Johnson MS (2004) The functional significance of individual variation in basal metabolic rate. Physiological and Biochemical Zoology 77: 900–915. 15674765 10.1086/427059

[66] Speakman JR, Ergon T, Cavanagh R, Reid K, Scantlebury DM, et al (2003) Resting and daily energy expenditures of free-living field voles are positively correlated but reflect extrinsic rather than intrinsic effects. Proceedings of the National Academy of Sciences of the United States of America 100: 14057–14062. 14615588 10.1073/pnas.2235671100 PMC283545

[67] Bech C, Langseth I, Moe B, Fyhn M, Gabrielsen GW (2002) The energy economy of the arctic-breeding Kittiwake (*Rissa tridactyla*): a review. Comparative Biochemistry and Physiology a-Molecular & Integrative Physiology 133: 765–770. 10.1016/s1095-6433(02)00153-8 12443932

[68] Emlen W, Segal DS, Mandell AJ (1972) Thyroid state - effects on presynaptic and postsynaptic central noradrenergic mechanisms. Science 175: 79–82. 4400216 10.1126/science.175.4017.79

